# Obstetric Violence from the Perspective of Heise’s Social–Ecological Model: A Scoping Review

**DOI:** 10.3390/healthcare14213797

**Published:** 2026-11-06

**Authors:** Jairo Daniel Mateus-Peña, Alix Yaneth Perdomo-Romero, Claudia Andrea Ramírez-Perdomo

**Affiliations:** Master’s Program in Nursing, Universidad Surcolombiana, Neiva 410010, Colombia; j.d.mateusp2002@gmail.com (J.D.M.-P.); alixyaneth.perdomo@usco.edu.co (A.Y.P.-R.)

**Keywords:** obstetric violence, labor, obstetric, natural childbirth, nursing, obstetric nursing, health personnel

## Abstract

**Background**: Obstetric violence ranges from verbal abuse to non-consensual physical interventions, violating women’s fundamental rights during childbirth. Despite its prevalence, it is often analyzed as an isolated instance of individual misconduct, neglecting the structural and cultural determinants that normalize it. **Objective**: To analyze the scientific literature on obstetric violence, examining its associated factors from the theoretical lens of Heise’s social–ecological model. **Method**: A scoping review was conducted following the methodology of Arksey and O’Malley. Scientific literature on obstetric violence published between 2015 and 2025 was identified in PubMed, SciELO, CINAHL, Scopus, ScienceDirect, MEDLINE, and Taylor & Francis. A Braun and Clarke deductive thematic analysis was applied to categorize the findings into the four levels of the Heise model: ontogenetic, microsystem, exosystem, and macrosystem. **Results**: A total of 48 articles from 23 countries was included, predominantly from Africa 13 (27%) and South America 12 (25%), with Ethiopia 6 (13%) and Brazil 5 (10%) standing out. The studies revealed 38 factors associated with obstetric violence, distributed across the ontogenetic level (18), microsystem level (8), exosystem level (10), and macrosystem level (2), demonstrating the multifactorial and intersectional nature of the issue, where institutional failures undermine maternal autonomy. **Conclusions**: Obstetric violence goes beyond poor individual care, emerging as a systemic and structural phenomenon. Using Heise’s model the factors were organized into dimensions, highlighting problems such as service overload and resource constraints that create environments associated with greater exposure to abuse among the most vulnerable women. Therefore, strategies are needed to address structural failures, anticipate violence, and ensure a respectful and safe childbirth experience.

## 1. Introduction

The World Health Organization (WHO) recommends that births take place in health facilities capable of providing timely care in the event of complications and of reducing maternal and neonatal mortality [1]. Normal childbirth is defined as a spontaneously initiated, low-risk physiological process that culminates in the birth of the baby and the expulsion of the placenta, leaving both mother and child in good condition [2,3].

Despite advances in maternal care, disrespectful practices and forms of undignified treatment persist during childbirth care, especially toward vulnerable groups. The biomedical model allows healthcare providers to control the childbirth process, exposing women to unnecessary interventions that become normalized through routine practice [1,4]. The inappropriate and excessive use of technologies or procedures leads to iatrogenic complications, which increases the rate of unjustified cesarean sections and instrumental deliveries, thereby creating conditions that may impede aspects of early mother–child bonding [5,6].

To address this issue, scholars developed the concept of obstetric violence (OV), which originated in Latin America to describe a form of violence against women in the context of sexual and reproductive health [7]. The scientific literature shows that childbirth experiences are frequently marked by abuse, neglect, abandonment, and the denial of autonomy—all of which are considered OV [8,9]. In low-income countries, more than one-third of women experience OV, with physical assault, shouting, and taunting being common [10]. WHO has acknowledged this issue by releasing statements affirming the universal right to dignified carefree from discrimination during pregnancy and childbirth [1].

The consequences of OV extend beyond childbirth, causing negative impacts on sexual and reproductive health, physical recovery, and the development of the bond with the newborn [7,11]. Furthermore, studies link the experience of obstetric violence to the onset of depressive symptoms, anxiety, and postpartum depression [12,13], which can lead to functional impairment and even fatal outcomes [12]. The response to this issue has been the promotion of humanized childbirth that respects the physiology, opinions, and needs of women and their families [2]. However, significant gaps persist regarding communication and the explanation of procedures, suggesting that OV remains a major international issue [9,14].

OV is influenced by multiple factors—including cultural, social, and personal—and therefore requires a thorough analysis of how these factors interact. Heise’s social–ecological model provides a theoretical framework for understanding the multifactorial nature of violence [15]. This model helps move beyond a reductionist view and posits that violence is the result of power dynamics and systemic failures that operate simultaneously across multiple dimensions. The model organizes these factors into four integrated dimensions: ontogenetic, microsystem, exosystem, and macrosystem [15,16]. When applied to OV, each dimension of the ecosystem reveals different perspectives on the phenomenon. The ontogenetic level focuses on biographical and socioeconomic characteristics; the microsystem represents the immediate clinical environment of the delivery room; the exosystem encompasses institutional functioning; and the macrosystem encompasses the nation’s cultural and sociopolitical values [15,16,17].

Although there has been extensive research, both quantitative and qualitative, on OV [18,19,20], and various instruments for measuring it have been validated [21,22,23], there is a need to synthesize the global evidence to identify the interaction among the risk factors. Therefore, this study aims to analyze scientific literature on OV by relating its associated factors through Heise’s social–ecological model. This scoping review seeks to identify gaps in knowledge regarding the systemic integration of OV factors, providing a basis for the design of future prevention-oriented tools in clinical and public health settings.

## 2. Materials and Methods

A scoping review was conducted following the methodological framework proposed by Arksey and O’Malley, consisting of five stages: (1) defining the research question; (2) identifying relevant studies; (3) study selection; (4) charting the data; and (5) compiling, summarizing, and reporting the results [24]. The Preferred Reporting Items for Systematic Reviews and Meta-Analyses: Extension for Scoping Reviews (PRISMA-ScR) checklist [25] was also followed.

The protocol was registered on the Open Science Framework (OSF) platform under the title: “Scientific production on obstetric violence worldwide: a scoping review” Published on December 9, 2024 [26]. Although the initial protocol aimed to broadly identify associated factors, the specific application of Heise’s social–ecological model was adopted during the data extraction phase.

### Research Question and Eligibility Criteria

The research question was: What does the scientific literature report about obstetric violence experienced by women during labor and delivery in healthcare facilities worldwide, and what are its associated factors and determinants characterized? We used the PCC strategy: Population: Women during labor and delivery; Concept: Obstetrical violence; Context: Childbirth care in healthcare facilities worldwide.

The inclusion criteria were as follows: original research articles related to OV—including those that directly document women’s experiences, as well as those that address the phenomenon from the observational perspective of healthcare personnel and doulas—published in peer-reviewed journals in English, Spanish, or Portuguese between 2015 and 2025. Although the primary population consists of women during childbirth, studies based on observations made by professionals were also considered eligible, as they provide essential data on the dynamics of the environment in which women’s experiences are directly shaped.

A comprehensive search was conducted between November 2025 and February 2026 in seven electronic databases: five subscription-based provided by Universidad Surcolombiana (Scopus, Taylor & Francis, ScienceDirect,) and (CINAHL, MEDLINE) accessed via the EBSCOhost and two open-access (PubMed, SciELO). The search strategy was developed using keywords from the DeCS and MeSH subject headings, combined with Boolean operators (OR, AND).

To systematically capture relevant literature, the search strategy was designed around four conceptual blocks connected. Within each block, related keywords and MeSH/DeCS terms were combined. The conceptual blocks were defined as follows: (1) terms related to childbirth (Parturition; Labor; Obstetric; Natural Childbirth; Obstetric Delivery Planning); (2) terms related to violence (Obstetric Violence; Violence Against Women); (3) terms related to scientific production (Research; Health Services Research); and (4) terms related to nursing and health personnel (Health Personnel; Nursing; Obstetric Nursing).

The inclusion of specific terms related to health services research and health personnel (Blocks 3 and 4) was a deliberate methodological choice to focus the review strictly on clinical interactions and organizational dynamics within healthcare facilities. While prominent terms in the global literature such as ‘mistreatment’ and ‘disrespect and abuse’ were not explicitly included as free-text keywords, they were targeted through the controlled vocabularies (DeCS/MeSH) linked to the broader concept of violence in healthcare settings. These conceptual blocks formed the theoretical basis for the specific search queries adapted for each database (Table 1).

After applying the filters, the references were exported to the Rayyan tool to remove duplicates. After that, the unique records underwent a screening process; the selection was conducted in two stages by the reviewers independently. The initial screening involved reviewing titles and abstracts to exclude studies that were irrelevant according to the inclusion criteria. This was followed by a full-text review: Potentially eligible articles were read in their entirety to confirm their inclusion. Disagreements among the reviewers were resolved through discussion and consensus.

Data extraction was performed using a matrix in Microsoft Excel^®^, developed by the research team. The variables extracted included: author(s), year of publication, country, study objective, methodological design, sample characteristics (explicitly distinguishing whether the primary data source was patient self-report, provider observation, or a mix of both), and main findings related to factors associated with OV. This distinction allowed the research team to contextualize the origin of the reported factors during the synthesis. A deductive thematic analysis was performed, following the approach of Braun and Clarke [27].

Data extraction for the analysis of factors associated with OV was performed independently by two reviewers; any conceptual discrepancy was resolved through discussion and consensus. In order to ensure systematic coding, clear definitions were established to categorize the factors into the four interrelated dimensions of Heise’s social–ecological model [15]. The ontogenetic level grouped the biological, sociodemographic, and clinical history factors inherent to the woman. The microsystem was defined as the immediate environment of the delivery room; although some factors have organizational roots, they were coded at this level because the reviewed literature describes them as the immediate triggers of stress that alter direct interactions between the patient and healthcare personnel. The exosystem was defined as the formal hospital framework and its organizational structures including protocols such as informed consent. Finally, the macrosystem encompassed global economic determinants and economy-driven health insurance models.

## 3. Results

A total of 4780 articles was identified. After applying the eligibility criteria and removing duplicates, 2151 articles remained, of which 48 were included in the analysis; the selection process is described in Figure 1.

The 48 articles included research from 23 countries. The continental distribution was Africa 13 (27%), South America 12 (25%), North America 9 (19%), Asia 8 (17%), Europe 4 (8%), and Oceania 2 (4%). Geographic attribution reflects the country where the empirical study was conducted; for international reviews and multinational studies, the attribution was based on the first author’s institutional affiliation.

They were published primarily in the following years: 2022 9 (19%), 2023 8 (17%), and 2024 6 (13%). In terms of methodological designs, 17 (35%) were quantitative, 16 (33%) were qualitative, 9 (19%) were literature reviews, and 6 (13%) used mixed methods.

The analysis of the scientific evidence identified 38 associated factors, distributed across the four dimensions of the socio-ecological model. The frequency recorded for each factor indicates the number of articles (out of a total of 48) in which it is mentioned. It should be noted that, although literature reviews have been included, they are treated as independent units of analysis. Since each review provides a unique synthesis and analysis their inclusion reflects the relevance of a given factor within various academic debates, rather than representing a duplication of primary data (Table 2). It is important to emphasize that not all of these factors are independently confirmed epidemiological risk factors; rather, they are identified as associated factors encompassing a broad spectrum of individual patient characteristics, hospital organizational dynamics, and specific clinical procedural conditions—all of which, according to the reviewed studies, are linked to the occurrence or perception of workplace violence (Table 3).

### 3.1. Dimension I

This dimension encompasses personal, obstetric, and sociodemographic characteristics that establish risk profiles prior to clinical interactions. The analysis shows that OV most frequently affects women who face multiple vulnerabilities, and that socioeconomic or educational privilege can act as a protective factor [29,35,38].

The findings suggest that healthcare professionals in the biomedical system may enforce norms around sexuality and reproduction. Adolescent girls face infantilization and interventions aimed at controlling childbirth [42,48,56], while older women are criticized for exceeding the recommended age for pregnancy [67,72]. Primiparous women undergo more interventions, and multiparous women suffer from clinical neglect based on the belief that they have mastered the birthing process [46,67]. Another risk factor involves pregnant women who fall outside traditional family models—such as single women, divorced women, or those belonging to LGBTIQ+ communities [38,47,54,73].

Also, being obese or overweight during pregnancy, having a disability, or testing positive for sexually transmitted infections such as HIV and syphilis increase the risk of OV [8,35,49,58]. Evidence shows that pregnant women with a history of domestic violence may be revictimized during childbirth [41,70]. Ethnic minorities may suffer from obstetric racism; women belonging to Indigenous groups, those of African descent, undocumented migrants, and those of non-traditional religious backgrounds experience higher rates of abuse [19,39,41,68] than women without these characteristics.

Additionally, women without health insurance face stigmatization that contributes to OV [40,57]. Some women with financial constraints that prevent them from obtaining basic supplies for their care are stigmatized, which contributes to OV that is often sustained through institutional silence and normalization of abusive practices [29,59]. Low levels of education and the absence of prenatal checkups are associated with greater vulnerability to OV; healthcare providers consider illiteracy and insufficient prenatal checkups to be sufficient reasons to intervene in the labor process [51,65]. Women with higher levels of education and greater knowledge about OV report these experiences more frequently [34,44,62,66].

Furthermore, pregnant women living in remote rural areas are more likely to face disrespectful and abusive care [65]. Finally, preexisting medical conditions increase the risk of OV; high-risk pregnancies lead to prioritizing interventions over humane care—an approach justified by the urgency of the situation [39,62]. The method of delivery is also a factor associated with the risk of OV. While unplanned or emergency interventions—such as urgent cesarean sections or instrumental deliveries—are often medically indicated to ensure the safety of both mother and fetus, they may be experienced or interpreted by the woman as OV if they are carried out without adequate communication and proper informed consent [62]. In such high-pressure situations, a patient’s decision-making capacity may be diminished by the urgency of the process; however, the medical intervention itself is not inherently considered violent or an act of OV. Rather, it is the lack of appropriate communication—or the unjustified disregard for a birth plan—that intensifies the perception of mistreatment. Evidence shows that a loss of control and the denial of autonomy lie at the root of OV in the delivery room [37,62].

### 3.2. Dimension II

The presence of a support network and a birth companion during labor is the primary protective factor against OV [19,42,61,62]. Even so, institutional bans on birth companions reach 64% in some contexts [33,71].

Likewise, social capital determines the quality of care: Knowing the medical staff beforehand is a strong protective factor [36], but at the same time it fosters patronage, where empathy and resources are reserved for acquaintances or those with referrals, potentially exposing other pregnant women to care that leaves them feeling neglected [33,63]. Communication barriers and lack of understanding of medical terminology foster relationships of subordination. Women who do not speak the country’s common language are more than twice as likely to receive negative treatment (aRR: 2.28 95% CI: (1.30 3.98)) [33], and failure to understand healthcare providers’ language and follow their instructions is perceived as disobedience, leading to hostile treatment by healthcare providers [29,32].

In addition, a break in continuity of care is another risk factor. Women referred to higher-level facilities are admitted as unknown patients, and such transfers increase the risk of intrapartum assault by more than three times (aOR = 3.7673, *p* = 0.030) [42], especially if the referral occurs following a failed attempt at home birth [39].

Finally, the microsystem of the delivery room is shaped by the resources provided by the institution. While staff shortages and excessive clinical workloads may have their roots in the exosystem, they often manifest as acute, unpredictable events in daily clinical practice. Sudden staff absences—such as those due to sick leave—or an unexpected surge in obstetric admissions cannot be precisely anticipated by institutional management. Consequently, these factors primarily act as immediate stressors within the microsystem, abruptly altering direct interactions between healthcare staff and the patient in labor. This unpredictability is described in the reviewed studies as a significant contributor to excessive workload and professional exhaustion [52]; some professionals are reported to develop chronic fatigue and burnout syndrome [40,45]. Faced with perceived pressure to free up beds and relieve congestion in the unit, accounts suggest healthcare staff frequently resort to accelerated labor management, typically using oxytocin, repeated vaginal examinations, and the Kristeller maneuver to speed up deliveries [53,72].

This problem reaches a critical level during the night shift; studies show that being in labor at night or in the early morning is an independent risk factor associated with inadequate care [48,67]. Staff fatigue and the lack of administrative oversight during these hours create a period of reduced accountability that facilitates OV [40,47].

### 3.3. Dimension III

This dimension examines the administrative and organizational structures of the healthcare system. At this level, qualitative accounts frequently interpret OV as a form of structural violence, where material and regulatory deficiencies are perceived to create an environment that compromises safe and dignified care [33,45].

Evidence shows that infrastructure deficiencies violate women’s privacy and dignity, especially in shared examination rooms [8,58], or when students are present without adequate information or consent, increasing feelings of shame and lack of privacy [35,60]. At the same time, shortages of supplies and equipment force patients into unsafe conditions during care, increasing workplace stress among healthcare professionals and contributing to inhumane behavior and verbal abuse [40,63,69].

In addition to physical barriers, rigid institutional policies—such as imposing the lithotomy position for logistical convenience, restricting movement, and depriving patients of their bodily autonomy—create conditions that facilitate OV [43,56,71]. This loss of autonomy is linked to the failure to obtain informed consent; women in public hospitals and those from ethnic minorities are more likely to undergo procedures without consent [30,53,68].

The context in which OV arises is shaped by a lack of ongoing training and institutional quality accreditation goals; the scarcity of training programs on patient-centered care and updated obstetric practices forces professionals to repeat outdated interventions and maintain inappropriate attitudes [42,51]. In contrast, hospitals with quality certifications significantly reduce the likelihood of OV [30].

Finally, the type of healthcare institution influences perceptions and care. Giving birth in the public healthcare system increases the likelihood of experiencing mistreatment compared to the private sector [41], due to the high volume of patients in public settings and the differences between the two patient populations [39,64]. Furthermore, public health facilities can perpetuate a culture of impunity: When reporting mechanisms are inaccessible and disciplinary sanctions are not enforced, power imbalances are reinforced, and healthcare professionals who commit OV face no accountability, leaving women to endure mistreatment in silence [8,19,63].

### 3.4. Dimension IV

This dimension addresses the outermost level of Heise’s ecological model, examining the sociopolitical, economic, and cultural architecture of society together with its healthcare system [15,69]. Obstetrical violence is linked to structural inequalities resulting from the commodification of health care. Evidence reveals that various healthcare systems operate under market-driven logic focused on sources of revenue and expenditure; the segmentation and stratification of services restrict universal equity, determining service priority and quality based on purchasing power [28,42,53]. This neoliberal structure leads to consequences such as the closure of delivery rooms because they are considered unprofitable, which overwhelms the remaining facilities [55] and institutionalizes OV by turning dignity into a class privilege [30,31].

This economic factor is linked to a country’s macroeconomic and geopolitical classification. Although OV is a global phenomenon, the literature confirms that abuse during childbirth is prevalent and severe primarily in low- and middle-income countries [37,58]. Living in low- or middle-income countries establishes a baseline of precariousness that permeates all levels, from the hospital ecosystem down to the clinical microsystem; resource limitations in underdeveloped countries heighten risk factors; marginalized women are more likely to suffer abuse in low-income countries compared to women in similar circumstances in high-income nations [49,58]. The eradication of OV requires a structural transformation that reduces or eliminates these deep-seated macrosystemic inequities in healthcare systems [28,40,42].

## 4. Discussion

Understanding OV as a systematic violation of women’s rights requires an in-depth analysis that goes beyond viewing it as a failure of individual care. Framing this phenomenon within the social–ecological model reveals how the interactions between factors across dimensions produce an ongoing pattern of heightened risk during pregnancy, and childbirth period [15].

The ontogenetic level allows us to examine how personal and socioeconomic characteristics influence the likelihood of experiencing or perceiving situations of OV in maternity wards [28,31]. The reviewed literature conclusively demonstrates that the risk of experiencing OV is not distributed equally but increases when multiple axes of vulnerability are present [45]. Certain aspects stand out. Belonging to an ethnic minority or being an undocumented migrant are critical factors for discrimination, as these pregnant women often face communication barriers and their ancestral knowledge is not recognized, reducing them to a subordinate position in care [46,55]. This is a global phenomenon; evidence from Europe confirms the persistence of obstetric racism toward ethnic minorities, which increases the risk of lower-quality care and more aggressive treatment [34,62].

However, an analysis of socioeconomic variables reveals a paradox: Some studies indicate that women with higher levels of education and greater access to information tend to report OV behaviors by healthcare personnel more frequently [34,73], but this does not mean that populations with lower levels of education suffer fewer instances of violence. On the contrary, a lack of knowledge about reproductive rights leads to underreporting in the most impoverished sectors [57,71]. This underreporting is known as the normalization of OV; it occurs when professionals fail to recognize such acts as OV, and also among the patients themselves, who internalize OV as inherent to childbirth, assuming that pain, reprimands, and the denial of their autonomy are the price they must pay for care, especially when it is free or subsidized [68,70,71].

The microsystem is where interactions between pregnant women and healthcare teams take place; in other words, it is the setting where OV most directly occurs, and where communication is key, yet studies agree that one of the most common forms of OV arises when healthcare personnel use fear of clinical outcomes as a manipulative tool to force acceptance of interventions that are neither consensual nor justified [33,38,62,70]. In this context, qualitative research highlights the phrase “I have to listen to them or they might hurt me,” which reflects a breakdown of trust and the establishment of a relationship of subordination [68,70,72].

From a clinical obstetric perspective, it is important to establish a distinction between OV practices and medically necessary interventions. As healthcare professionals, we know that in daily practice, especially during obstetric emergencies, rapid decision-making—such as an emergency cesarean section or an instrumental delivery—is vital to prevent maternal and fetal complications, and these actions do not constitute OV. The literature demonstrates that the line defining OV does not lie in the clinical intervention itself, but in the circumstances surrounding it; therefore, clear communication must remain a priority even in emergency scenarios [62,68,72].

In light of this microsystem problem, studies reveal that the presence of a continuous birth companion during childbirth acts as a protective factor; the emotional support provided by family members reduces the incidence of abuse of any kind and of unnecessary procedures [47,54,61]. The birth companion also acts as a witness in the delivery room, limiting instances of disrespectful behavior [36,41]. However, some hospital microsystems resist allowing companions, citing administrative arguments that mask a desire to maintain control over the birth process [39,42].

As the analysis moves toward the exosystem, it disassociates OV from individual care and frames it as a consequence of organizational deficiencies in the healthcare system [69]. Overburdened institutional settings, characterized by staff shortages, a lack of basic supplies, and deteriorating infrastructure, contribute to dehumanizing treatment [31,40,45,52]. Multiple studies describe how healthcare personnel, exposed to long workdays and high levels of burnout, develop a hostile attitude as a defense against institutional collapse and chaos [33,38,40,59]. In this ecosystem, healthcare staff prioritize the efficiency of patient flow over the quality of care, and invasive and routine procedures are rushed to free up beds, ensuring the capacity to accommodate and attend to other deliveries [44,60].

Additionally, the lack of internal oversight mechanisms and the absence of channels for reporting abuse institutionalize impunity within hospitals, reinforcing the normalization of OV, and the right to a respectful birth is viewed as utopian rather than as a mandatory standard [65,72].

Cultural values lie at the macrosystem level, and consist of political ideologies, gender roles, and economic models that underpin OV globally. Obstetrical violence cannot be separated from the biomedical model adopted by the country, especially when the healthcare system is structured as a market commodity [45]. Macroeconomic policies of privatization and commodification of health services undermine the quality of care, widening the inequality gap and forcing disadvantaged women to receive fragmented care that lacks quality standards [57,69]. The global persistence of OV demonstrates that the phenomenon stems from a structural framework resulting from the devaluation of care work and the dehumanization of bodies under institutional control [62,63,69].

Therefore, the analysis of obstetric violence goes beyond clinical interactions and must be placed within a broader context of deep-seated inequalities, organizational problems, and barriers to access to medical care; eradicating these structural and systemic failures requires holistic solutions. As recent evidence indicates, protecting maternal health and reducing morbidity and mortality is a global multidisciplinary imperative [74]. Only through a collaborative and multidisciplinary approach that goes beyond the traditional biomedical model will it be possible to transform health policies, mitigate social inequities, and restructure institutional environments to ensure safe and respectful maternal care.

This research presents limitations. First, there is significant methodological heterogeneity among the included studies, featuring a wide variation in study designs, populations, and healthcare systems, which limits the comparability and direct generalizability of the findings. Second, geographical differences are evident; the evidence is predominantly concentrated in certain regions, leaving a gap in understanding the phenomenon in other global contexts. Third, the normalization of OV in many settings frequently leads to underreporting, which may skew the documented prevalence and the identification of associated factors.

Fourth, there are recognized limitations in the search strategy. The use of restrictive disciplinary terms—such as “nursing,” “health personnel,” and “health services research”—was intended to focus the review on clinical and organizational dynamics; however, this decision may have inadvertently excluded relevant studies from other perspectives. Furthermore, relying primarily on controlled vocabularies (MeSH/DeCS) without explicitly incorporating free-text synonyms—such as “mistreatment” or “lack of respect and abuse”—into the search strings likely reduced the sensitivity of the search. Additionally, the use of multiple Boolean operators (AND, OR) without strict grouping via parentheses in some of the search strings (Table 1) may have also affected precision. Finally, by restricting the results to articles with full text available, studies requiring payment may have been excluded, meaning that a portion of the overall evidence is not represented in the final analysis.

Fifth, the adoption of Heise’s socio-ecological model during the data extraction phase has implications for the interpretation of our findings, as it necessitated a retrospective categorization of data from studies that were not originally framed within an ecological model. Consequently, this may limit the depth of the systemic interactions described, given that the primary sources did not capture the multidimensional complexity inherent in the ecological model.

## 5. Conclusions

An analysis of obstetrical violence using Heise’s social–ecological model highlights that OV is a multifactorial phenomenon. It transcends the notion of simply being poor conduct on the part of healthcare personnel, and is better understood as a form of systemic, structural, and gender-based violence that often permeates the social system where it occurs. Heise’s model allows for the breakdown and organization of the multiple factors associated with OV, revealing that it is closely linked to violation of rights, which is exacerbated by interactions between sociodemographic marginalization, power asymmetries within institutions, administrative precariousness, and the logic of the healthcare system.

According to the synthesized literature, healthcare systems driven by economic priorities and social stratification are frequently described by participants as fostering environments characterized by inequality, overcrowding, shortages, work overload, and limited accountability. Rather than establishing direct causality, the aggregated qualitative and observational evidence suggests that in such exhausting contexts, professional burnout is strongly associated with the displacement of stress onto vulnerable patients during childbirth—a dynamic that is further compounded when a birth companion is prohibited.

This review provides a useful conceptual foundation for the design of future assessment tools, intervention programs, and strategies aimed at promoting respectful, safe, and women’s rights-centered maternal care. In terms of clinical implications, the findings emphasize the need to strictly enforce informed consent protocols and maintain clear, empathetic communication throughout labor, ensuring women remain active participants in their care even during obstetric emergencies. Organizationally, healthcare facilities must implement practical measures such as optimizing staffing levels to mitigate burnout, guaranteeing the continuous presence of birth companions, and establishing transparent reporting mechanisms to improve respectful maternity care.

Furthermore, future research should explore in depth the psychological consequences of these negative experiences—such as postpartum anxiety and depression—and assess the psychological support needed for women affected by OV.

## Figures and Tables

**Figure 1:**
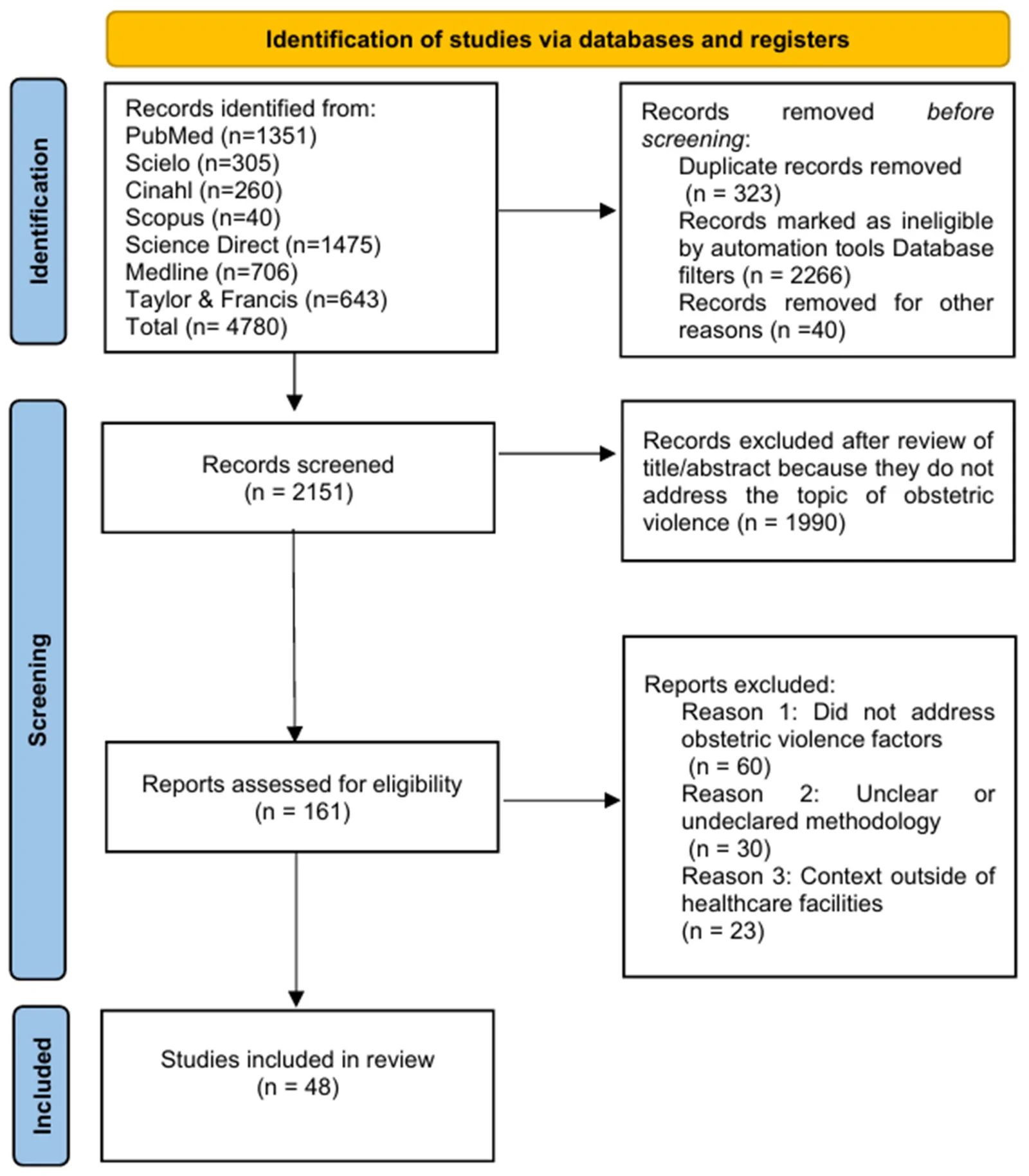
Flowchart of the article selection process.

**Table 1:** Search queries for each database.

Databases/Last Search Date	Search Queries	Filters Applied
PubMed 20 December 2025	Search 1: “Obstetric Violence” AND Parturition OR “Labor, Obstetric” AND “Health Personnel” OR “Obstetric Nursing” AND Research.	Full text, English, Portuguese, Spanish, from 2015 to 2025
	Search 2: Obstetric Delivery Planning OR Parturition OR Natural Childbirth AND Obstetric Violence OR Violence Against Women AND Nursing AND Health Services Research	
Scielo 22 January 2026	Search 1: ((Obstetric Violence) AND (Parturition) OR (Labor, Obstetric)) AND (Health Personnel) OR (Obstetric Nursing)	Languages: Portuguese, English, and Spanish. (Publication year: 2015–2025) (Literature type: Article, Review article)
	Search 2: (((((((Obstetric Delivery Planning)) OR (Parturition)) OR (Natural Childbirth)) AND (Obstetric Violence)) OR (Violence Against Women)) AND (Health Services Research)) AND (Nursing)	
Cinahl 23 January 2026	Search 1: XB (Obstetric Violence AND Parturition OR Labor, Obstetric AND Health Personnel OR Obstetric Nursing AND Research)	English; Portuguese; Spanish Full text Peer-reviewed publications 1 January 2015–31 December 2025
	Search 2: TX obstetric delivery planning OR TX (parturition and obstetric violence) OR TX natural childbirth AND TX (violence against women or gender-based violence or domestic violence or domestic abuse or intimate partner violence) AND nursing AND health services research	
Scopus 23 January 2026	Search 1: (“Obstetric Violence” AND parturition OR “Labor, Obstetric” AND “Health Personnel” OR “Obstetric Nursing” AND research)	TITLE-ABS-KEY AND PUBYEAR > 2014 AND PUBYEAR < 2026
	Search 2: (“Obstetric Delivery Planning” OR parturition OR “Natural Childbirth” AND “Obstetric Violence” OR “Violence Against Women” AND “Health Services Research” OR nursing)	
Science Direct 21 January 2026	Search 1: “Obstetric Violence” AND “Parturition” OR “Labor, Obstetric” AND “Health Personnel” OR “Obstetric Nursing” AND “Research”	Years: 2015–2025 Article type: Review Articles–Research Article Languages: English–Spanish
	Search 2: Title: “Obstetric Delivery Planning” OR “Parturition” OR “Natural Childbirth” AND “Obstetric Violence” OR “Violence Against Women” AND “Nursing” AND “Health Personnel”	
Medline 25 January 2026	Search 1: Obstetric Violence AND Parturition OR Labor, Obstetric AND Health Personnel OR Obstetric Nursing AND Research	English; Portuguese; Spanish Full text Peer-reviewed publications 1 January 2015–31 December 2025
	Search 2: TX obstetric delivery planning OR TX (parturition and obstetric violence) OR TX natural childbirth AND TX (violence against women or gender-based violence or domestic violence or domestic abuse or intimate partner violence) AND nursing AND health services research	
Taylor & Francis 25 January 2026	Search 1: “Obstetric Violence” AND Parturition OR “Labor, Obstetric” AND “Health Personnel” OR “Obstetric Nursing” AND Research	[Article Type: Article] AND [Publication Date: (1 January 2015 TO 31 December 2025)]
	Search 2: Obstetric Delivery Planning OR Parturition OR Natural Childbirth AND “Obstetric Violence” OR “Violence Against Women” AND Nursing AND Health Services Research	

**Table 2:** Factors identified in scientific literature.

Dimension	Ecological Level	Factors/(Number of Articles Between 48)
Dimension I	Ontogenetic	(1) Maternal age (23); (2) Health insurance status (8); (3) Personal finances (24); (4) Marital status (13); (5) Socioeconomic status (17); (6) Education level (18); (7) Place of residence (11); (8) Religion (4); (9) Ethnicity (19); (10) Hygiene kit (4); (11) History of violence (6); (12) Knowledge of OV (8); (13) Vulnerability (20); (14) Prenatal checkups (7); (15) Number of births (19); (16) High obstetric risk (4); (17) Nutritional status (4); (18) Planned method of delivery (8).
Dimension II	Microsystem	(1) Support (7); (2) Communication barriers (4); (3) Support network (5); (4) Human Resources (8); (5) Continuity of care (5); (6) Staff knowledge (3); (7) Service saturation (9); (8) Time of delivery (5).
Dimension III	Exosystem	(1) Privacy (9); (2) Type of facility (4); (3) Presence of students (3); (4) Supplies and equipment (7); (5) Quality accreditation (2); (6) Training (4); (7) Birthing positions (9); (8) Consent (8); (9) Methods of reporting (4); (10) Sanctions (3).
Dimension IV	Macrosystem	(1) System structure (4); (2) Country classification (3).

**Table 3:** Factors identified in the scientific literature in each included article.

Titles/(Year)/Reference	Study Objective/(Database)	Dimension/Identified Factors
Neither medicine nor health care staff members are violent by nature: Obstetric violence from an interactionist perspective. (2018) [28]	To understand the meaning women attribute to healthcare practices performed during childbirth. (PubMed)	Dimension I: Maternal age; Personal finances; History of violence; Knowledge of OV; Nutritional status.
“If we’re here, it’s only because we have no money.” Discrimination and violence in Mexican maternity wards. (2018) [29]	To analyze women’s experiences of structural and gender-based discrimination during childbirth care in two public hospitals in Mexico. (PubMed)	Dimension I: Maternal age; Health insurance status; Personal finances; Socioeconomic status; Education level; Knowledge of OV; Number of births.
Bearing witness: United States and Canadian maternity support workers’ observations of disrespectful care in childbirth. (2018) [30]	To compare reports from birth doulas and labor and delivery nurses regarding witnessing disrespectful care in the United States and Canada. (PubMed)	Dimension II: Human Resources; Service saturation. Dimension III: Quality accreditation; Consent. Dimension IV: System structure.
Evidence of ‘obstetric violence’ in India: An integrative review. (2020) [31]	To analyze the literature on OV in India using Bohren’s comprehensive typology and highlight findings that do not align with Bohren’s framework in order to identify all types of obstetric violence in India and compare them with global findings. (PubMed)	Dimension I: Personal finances; Socioeconomic status; Hygiene kit; Vulnerability; Number of births. Dimension II: Human Resources; Service saturation. Dimension III: Privacy; Type of facility. Dimension IV: System structure.
Birth experience in Syrian refugee women in Turkey: A descriptive phenomenological qualitative study. (2021) [32]	To provide an in-depth description of the childbirth experiences of Syrian refugee women in Turkey. (PubMed)	Dimension I: Religion; Ethnicity; Vulnerability. Dimension II: Communication barriers; Support network.
An exploratory study of client and provider experience and perceptions of facility-based childbirth care in Quiché, Guatemala. (2022) [33]	To analyze women’s negative and positive experiences with childbirth care, as well as the perceptions and experiences of healthcare personnel in providing maternal care. (PubMed)	Dimension I: Maternal age; Marital status; Education level; Ethnicity; Number of births; High obstetric risk.
Disrespect and abuse during labour and birth amongst 12,239 women in the Netherlands: A national survey. (2022) [34]	To examine the frequency with which women experience disrespect and abuse during labor and birth in the Netherlands and the frequency with which they consider these experiences distressing. (PubMed)	Dimension I: Maternal age; Education level; Ethnicity; Vulnerability; Number of births.
Dehumanized, violated, and powerless: An Australian survey of women’s experiences of obstetric violence in the past 5 years. (2024) [35]	To explore experiences of OV reported in a 2021 national survey of Australian women who had given birth in the past 5 years. (PubMed)	Dimension I: Maternal age; Personal finances; Ethnicity; Nutritional status.
Prevalence and risk factors of obstetric violence in the Gaza strip: A retrospective study from a conflict setting. (2023) [36]	To assess the prevalence, determinants, and common types of verbal abuse among women who gave birth in the past four years at various hospitals in the Gaza Strip. (PubMed)	Dimension I: Place of residence; Knowledge of OV; Number of births.
Experienced disrespect & abuse during childbirth and associated birth characteristics: A cross-sectional survey in the Netherlands. (2024) [37]	To explore the frequency with which OV is experienced in the Netherlands and considered distressing, and which birth characteristics are associated with these distressing experiences of OV. (PubMed)	Dimension I: Number of births; Planned method of delivery.
“Is the doctor God to punish me?!” An intersectional examination of disrespectful and abusive care during childbirth against single mothers in Tunisia. (2017) [38]	To examine the self-perceptions and childbirth experiences of single mothers in public health centers in Tunisia. (PubMed)	Dimension I: Personal finances; Marital status; Socioeconomic status; Education level
The giving voice to mothers study: Inequity and mistreatment during pregnancy and childbirth in the United States. (2019) [39]	To present the indicators of abuse designed by patients that align with the typology proposed by Bohren, where relevant to service users in high-resource settings, and to examine the relationships between race and abuse in the context of factors frequently associated with health inequity. (PubMed)	Dimension I: Maternal age; Health insurance status; Personal finances; Socioeconomic status; Place of residence; Ethnicity; History of violence; Vulnerability; Number of births; High obstetric risk; Planned method of delivery.
Providers’ perceptions of disrespect and abuse during childbirth: A mixed-methods study in Kenya. (2020) [40]	To examine providers’ perspectives on the frequency and drivers of disrespect and abuse during childbirth at a health facility in a rural county in Kenya. (PubMed)	Dimension I: Maternal age; Personal finances; Socioeconomic status; Hygiene kit; Number of births.
Obstetric violence is prevalent in routine maternity care: A cross-sectional study of obstetric violence and its associated factors among pregnant women in Sri Lanka’s Colombo District. (2022) [41]	To investigate the possible association between domestic violence and the experience of OV, as well as any significant relationship between the occurrence of obstetric violence and the reporting of domestic violence among pregnant women in Sri Lanka. (PubMed)	Dimension I: Maternal age; Personal finances; Education level; Place of residence; Ethnicity; History of violence; Vulnerability.
Intrapartum violence during facility-based childbirth and its determinants: A cross-sectional study among postnatal women in Tanzania. (2023) [42]	To assess the types of intrapartum violence and their determinants among postnatal women in the Dodoma region of Tanzania. (PubMed)	Dimension I: Personal finances; Place of residence; Prenatal checkups; Number of births. Dimension II: Support; Continuity of care.
‘It was horrible to watch, horrible to be a part of’: Midwives’ perspectives on obstetric violence. (2024) [43]	To explore Australian midwives’ perspectives on obstetric violence. (PubMed)	Dimension I: Ethnicity. Dimension II: Continuity of care. Dimension III: Presence of students; Birthing positions.
Obstetric violence and associated factors among women who gave birth at public hospitals in Addis Ababa city administration, Ethiopia. (2024) [44]	To assess the prevalence of obstetric violence and associated factors among women who gave birth in public hospitals under the Addis Ababa City Administration, Ethiopia. (PubMed)	Dimension I: Education level; Knowledge of OV; Prenatal checkups; Planned method of delivery.
Layers of inequality: Gender, medicalization, and obstetric violence in Ghana. (2024) [45]	To examine gender inequalities in health systems and explore how they contribute to women’s experiences of OV. (PubMed)	Dimension I: Marital status; Vulnerability. Dimension II: Human resources; Service saturation. Dimension III: Privacy; Supplies and equipment; Training.
Una mirada desde la interseccionalidad a la violencia obstétrica en mujeres indígenas (An intersectional perspective on obstetric violence among Indigenous women) (2023) [46]	To analyze the experiences and conditions that contributed to vulnerability among a group of Nahua women in the context of obstetric violence. (Scielo)	Dimension I: Health insurance status; Personal finances; Socioeconomic status; Place of residence; Ethnicity; History of violence. Dimension II: Communication barriers.
Violência obstétrica: revisão sistemática internacional (Obstetric violence: An international systematic review) (2023) [47]	To conduct an international systematic review on obstetric violence (OV) using the PRISMA method. (Scielo)	Dimension I: Personal finances; Marital status; Socioeconomic status; Education level; Vulnerability. Dimension II: Support; Support network; Continuity of care. Dimension III: Quality accreditation; Birthing positions; Consent.
Percepción de atención obstétrica inadecuada a pacientes de un hospital público de Ciudad Juárez, Chihuahua (Perceptions of inadequate obstetric care among patients at a public hospital in Ciudad Juárez, Chihuahua) (2023) [48]	To determine patients’ perceptions of mistreatment or actions contrary to standard practice during obstetric care and to identify the factors associated with these perceptions among patients treated at a public hospital in Ciudad Juárez, Chihuahua. (Scielo)	Dimension I: Maternal age; Education level; Number of births. Dimension II: Time of delivery
Violencia obstétrica capacitista hacia mujeres con discapacidad: Una revisión integradora de la literatura (Ableist obstetric violence against women with disabilities: A comprehensive literature review) (2023) [49]	To identify and analyze the literature on obstetric violence against women with disabilities. (Scielo)	Dimension I: Vulnerability; Number of births. Dimension IV: Country classification.
Racismo obstétrico em Portugal: Relato de experiência de um coletivo antirracista (Obstetric racism in Portugal: An anti-racist collective’s experience report) (2022) [50]	To report on the experiences of this collective of active anti-racist women in Portugal from an intersectional perspective. (Scielo)	Dimension I: Personal finances; Ethnicity.
Obstetric violence: A conceptual analysis in the nursing context (2022) [51]	To analyze the concept of “OV” in the nursing context by identifying its background, attributes, and consequences. (Scielo)	Dimension I: Health insurance Status; Personal finances; Socioeconomic status; Education level; Ethnicity; Knowledge of OV; Vulnerability; Prenatal checkups. Dimension III: Training; Birthing positions; Consent.
Obstetric violence in childbirth care in Baja California: Nursing staff perceptions (2022) [52]	To analyze nursing staff perceptions of abuse against women during pregnancy and childbirth. (Scielo)	Dimension I: Maternal age; Vulnerability; Prenatal checkups; Number of births; Nutritional status. Dimension II: Human resources; Service saturation. Dimension III: Privacy; Supplies and equipment; Training.
Violencia obstétrica en Chile: percepción de las mujeres y diferencias entre centros de salud (Obstetric violence in Chile: Women’s perceptions and differences among health centers) (2022) [53]	To report the results of the first survey on obstetric violence in Chile, in order to shed light on a reality that is more common than we realize and to compare its occurrence according to the type of service (public or private) where the delivery took place. (Scielo)	Dimension I: Maternal age; Vulnerability. Dimension II: Service saturation. Dimension III: Type of facility; Consent. Dimension IV: System structure.
Da violência obstétrica ao empoderamento de pessoas gestantes no trabalho das doulas (From obstetric violence to the empowerment of pregnant people in the work of doulas) (2021) [54]	To understand the meaning, they attribute to their experiences and lived realities in their work as doulas, as well as the relational contexts in which they operate. (Scielo)	Dimension I: Maternal age; Personal finances; Marital status; Socioeconomic status; Education level; Place of residence; Ethnicity; Vulnerability; Prenatal checkups.
“Parir no es un asunto de etnia, es un asunto de humanidad”: experiencias frente a la violencia obstétrica durante la atención al parto en mujeres indígena” (“Childbirth is not a matter of ethnicity, it is a matter of humanity”: Experiences with obstetric violence during childbirth care among Indigenous women) (2021) [55]	To understand the experiences of obstetric violence faced by “Embera” women during childbirth care at health facilities in the city of Medellín, Colombia. (Scielo)	Dimension I: Personal finances; Ethnicity. Dimension II: Communication barriers. Dimension IV: System structure.
“Violencia obstétrica: haciendo visible lo invisible” (Obstetric violence: making the invisible visible) (2019) [56]	To characterize the different forms of OV. (Scielo)	Dimension I: Maternal age; Number of births. Dimension II: Human resources. Dimension III: Privacy; Birthing positions.
“A person who does not have money does not enter”: a qualitative study on refugee women’s experiences of respectful maternity care (2022) [57]	To present the experiences, understanding, and interpretation of disrespectful treatment and abuse during childbirth in Lebanese hospitals by Palestinian, Iraqi, and Syrian refugee women. (Scopus)	Dimension I: Health insurance status; Personal finances; Socioeconomic status; Vulnerability.
Obstetric violence and disability overlaps: obstetric violence during childbirth among women with disabilities: A qualitative study (2022) [58]	To explore the experiences of women with disabilities regarding obstetric violence during childbirth in the Gedio region of southern Ethiopia, (Scopus)	Dimension I: Vulnerability. Dimension II: Communication barriers; Human resources. Dimension III: Privacy. Dimension IV: Country classification.
Disrespectful intrapartum care during facility-based delivery in sub-Saharan Africa: A qualitative systematic review and thematic synthesis of women’s perceptions and experiences. (2016) [59]	To examine women’s experiences with facility-based deliveries in sub-Saharan Africa to analyze the factors driving disrespectful intrapartum care. (ScienceDirect)	Dimension I: Personal finances; Marital status; Socioeconomic status; Place of residence; Hygiene kit Dimension II: Staff knowledge. Dimension III: Presence of students.
“Normal in all the rush”: A phenomenological study analyzing midwifery students’ views on obstetric violence (2024) [60]	To investigate midwifery students’ perceptions of OV. (ScienceDirect)	Dimension I: Health insurance status; Personal finances; Socioeconomic status; Ethnicity; Vulnerability. Dimension III: Privacy; Presence of students.
The prevalence of obstetric violence experienced by women during childbirth care and its associated factors in Türkiye: A cross-sectional study (2023) [61]	To determine the prevalence of obstetric violence experienced by women during childbirth and related factors in Türkiye. (ScienceDirect)	Dimension I: Maternal age; Marital status; History of violence; Prenatal checkups; Number of births; High obstetric risk. Dimension II: Support Dimension III: Birthing positions.
The magnitude of the problem of obstetric violence and its associated factors: A cross-sectional study (2021) [62]	To determine the prevalence of obstetric violence in the Spanish healthcare system and identify associated factors. (ScienceDirect)	Dimension I: Maternal age; Marital status; Education level; Knowledge of OV; Number of births; High obstetric risk; Planned method of delivery. Dimension II: Support; Support network. Dimension III: Birthing positions.
A qualitative analysis of obstetric violence in rural Madagascar (2023) [63]	To examine perceptions of the quality of care in rural areas, identifying women’s experiences and expectations regarding basic and emergency obstetric care, and how providers are meeting them. (ScienceDirect)	Dimension I: Socioeconomic status; Education level; Hygiene kit; Prenatal checkups. Dimension II: Staff knowledge. Dimension III: Supplies and Equipment; Methods of reporting.
Magnitude and associated factors of disrespect and abusive care among laboring mothers at public health facilities in Borena District, South Wollo, Ethiopia. (2021) [19]	To analyze the disrespect and abuse experienced by women in the delivery rooms of health facilities in Borena District, South Wollo, Ethiopia. (Medline)	Dimension I: Personal finances; Socioeconomic status; Vulnerability. Dimension II: Support Dimension III: Methods of reporting.
Disrespect and abuse during childbirth in Western Ethiopia: Should women continue to tolerate? (2019) [64]	This study presents the prevalence and factors associated with disrespect and abuse (D&A) during childbirth in public health facilities in Western Oromia, Ethiopia. (Medline)	Dimension I: Personal finances; Knowledge of OV. Dimension II: Support; Support network. Dimension III: Type of facility; Methods of reporting.
Disrespect and abuse during childbirth in Ethiopia: A systematic review. (2020) [65]	To synthesize the evidence on the scope, contributing factors, and consequences of disrespectful and abusive intrapartum care from the perspective of women and healthcare professionals in Ethiopia. (Medline)	Dimension I: Socioeconomic status; Education level; Place of residence; Religion; Ethnicity; Knowledge of OV; Vulnerability Dimension III: Sanctions.
The mistreatment of women during childbirth in health facilities globally: A mixed-methods systematic review. (2015) [8]	To synthesize qualitative and quantitative evidence on the mistreatment of women during childbirth in health facilities to inform the development of an evidence-based typology of the phenomenon. (Medline)	Dimension I: Maternal age; Marital status; Education level; Place of residence; Ethnicity; Vulnerability; Number of births. Dimension II: Support network; Service saturation. Dimension III: Privacy; Supplies and equipment; Training; Sanctions.
Midwives’ perspectives on (dis)respectful intrapartum care during facility-based delivery in Sub-Saharan Africa: A qualitative systematic review and meta-synthesis. (2019) [66]	To explore the broader factors driving (dis)respectful care during labor in health facilities in the context of sub-Saharan Africa. (Medline)	Dimension I: Maternal age; Education level; Place of residence; Ethnicity. Dimension III: Supplies and equipment; Birthing positions; Sanctions.
Obstetric violence in the multinational context. (2018) [67]	To review the scientific literature on OV. (Cinahl)	Dimension I: Maternal age; Personal finances; Socioeconomic status; Education level; Place of residence; Religion; Ethnicity. Dimension II: Time of delivery Dimension III: Privacy
Narratives of mistreatment and coercion in maternity care: An interpretive description and qualitative analysis. (2025) [68]	To describe and classify mistreatment during maternity care, as reported by a diverse group of women across the United States. (PubMed)	Dimension I: Health insurance status; Personal finances; Marital status; Ethnicity; Vulnerability; Number of births; Nutritional status; Planned method of delivery. Dimension II: Human resources; Staff knowledge; Service saturation; Time of delivery. Dimension III: Supplies and equipment; Consent.
How health system failures and prevailing social norms drive mistreatment of women in maternity care in Ethiopia: A qualitative study. (2025) [69]	To explore how deficiencies in the healthcare system and social norms that discriminate against women contribute to the mistreatment women experience during maternity care. (PubMed)	Dimension I: Maternal age Dimension II: Human resources; Continuity of care; Service saturation; Time of delivery. Dimension III: Privacy; Supplies and equipment; Training. Dimension IV: Country classification.
“I have to listen to them, or they might harm me” and other narratives of why women endure obstetric violence in Bihar, India (2025) [70]	To identify the factors driving OV during labor and delivery in Bihar, India. (PubMed)	Dimension I: Maternal age; Personal finances; Marital status; Socioeconomic status; Education level; Religion; History of violence; Number of births; Planned method of delivery. Dimension II: Support; Time of delivery Dimension III: Type of facility; Consent; Methods of reporting.
Percepción y experiencia de violencia obstétrica en puérperas de un hospital público en Perú: un estudio mixto (Perception and experience of obstetric violence among postpartum women at a public hospital in Peru: A mixed-methods study) (2025) [71]	To determine the perception and experience of OV among postpartum women during childbirth at a public hospital in Peru. (Scielo)	Dimension I: Maternal age; Planned method of delivery. Dimension II: Continuity of care; Service saturation. Dimension III: Birthing positions; Consent.
Obstetric violence in the State of Rio de Janeiro: Nascer no Brasil II Study (2025) [72]	To estimate the prevalence and comorbidity profile of OV among women hospitalized for childbirth in the state of Rio de Janeiro, as well as its distribution according to demographic, socioeconomic, and current pregnancy-related characteristics. (Scielo)	Dimension I: Maternal age; Health insurance status; Personal finances situation; Marital status; Education level; Ethnicity; Number of births; Planned method of delivery. Dimension III: Consent.
Percepción de violencia obstétrica y calidad de la atención en la región del maule, Chile (Perceptions of obstetric violence and quality of care in the Maule region, Chile) (2025) [73]	To understand the levels of OV perceived by a sample of women in the Maule Region, Chile, and to link them to perceptions of the quality of care. (Scielo)	Dimension I: Maternal age; Marital status; Vulnerability. Dimension III: Birthing positions.

## Data Availability

The data are available using the search equations described, and the RISK files can also be downloaded from the search protocol available on OSF: https://doi.org/10.17605/OSF.IO/J7FWG (accessed on 20 October 2026).

## Abbreviations

The following abbreviations are used in this manuscript:

DeCS	Descritores em Ciências da Saúde
HIV	Human Immunodeficiency Virus
LGBTIQ+	lesbian, gay, bisexual, transgender, queer, and other identities.
MeSH	Medical Subject Headings
PCC	Population, Concept, and Context.
OSF	Open Science Framework
OV	Obstetric violence
WHO	World Health Organization
